# Identification and prediction of novel classes of long-term disease trajectories for patients with juvenile dermatomyositis using growth mixture models

**DOI:** 10.1093/rheumatology/keaa497

**Published:** 2020-11-04

**Authors:** Claire T Deakin, Charalampia Papadopoulou, Liza J McCann, Neil Martin, Muthana Al-Obaidi, Sandrine Compeyrot-Lacassagne, Clarissa A Pilkington, Sarah L Tansley, Neil J McHugh, Lucy R Wedderburn, Bianca L De Stavola, for the Juvenile Dermatomyositis Research Group

**Affiliations:** 1 Infection, Immunity and Inflammation Research and Teaching Department, UCL Great Ormond Street Institute of Child Health, UCL, London, UK; 2 Centre for Adolescent Rheumatology Versus Arthritis at UCL, UCL Hospitals and Great Ormond Street Hospital, London, UK; 3 NIHR Biomedical Research Centre at Great Ormond Street Hospital, London, UK; 4 Rheumatology Unit, Great Ormond Street Hospital, London, UK; 5 Department of Paediatric Rheumatology, Alder Hey Children’s NHS Foundation Trust, Liverpool, UK; 6 Rheumatology Department, Royal Hospital for Sick Children, Glasgow; 7 Department of Pharmacy and Pharmacology, University of Bath, Bath, UK; 8 Population, Policy and Practice Research and Teaching Department, UCL Great Ormond Street Institute of Child Health, London, UK

**Keywords:** JDM, myositis, paediatric rheumatology, patient outcomes, biostatistics

## Abstract

**Objectives:**

Uncertainty around clinical heterogeneity and outcomes for patients with JDM represents a major burden of disease and a challenge for clinical management. We sought to identify novel classes of patients having similar temporal patterns in disease activity and relate them to baseline clinical features.

**Methods:**

Data were obtained for *n* = 519 patients, including baseline demographic and clinical features, baseline and follow-up records of physician’s global assessment of disease (PGA), and skin disease activity (modified DAS). Growth mixture models (GMMs) were fitted to identify classes of patients with similar trajectories of these variables. Baseline predictors of class membership were identified using Lasso regression.

**Results:**

GMM analysis of PGA identified two classes of patients. Patients in class 1 (89%) tended to improve, while patients in class 2 (11%) had more persistent disease. Lasso regression identified abnormal respiration, lipodystrophy and time since diagnosis as baseline predictors of class 2 membership, with estimated odds ratios, controlling for the other two variables, of 1.91 for presence of abnormal respiration, 1.92 for lipodystrophy and 1.32 for time since diagnosis. GMM analysis of modified DAS identified three classes of patients. Patients in classes 1 (16%) and 2 (12%) had higher levels of modified DAS at diagnosis that improved or remained high, respectively. Patients in class 3 (72%) began with lower DAS levels that improved more quickly. Higher proportions of patients in PGA class 2 were in DAS class 2 (19%, compared with 16 and 10%).

**Conclusion:**

GMM analysis identified novel JDM phenotypes based on longitudinal PGA and modified DAS.

**Rheumatology key messages**

- Sub-phenotypes of JDM based on patient outcomes have not previously been well defined.
- Description of sub-phenotypes of JDM based on global and skin disease activities over time.
- Abnormal respiration, lipodystrophy and more time since diagnosis at baseline predict severe disease in JDM.

## Introduction

Uncertainty around medium- to long-term outcomes is a major burden of disease for patients and families, as highlighted by a recent qualitative study of children and young people with JDM [1]. In addition to characteristic features of proximal muscle weakness and pathognomonic skin rash, many patients develop a heterogeneous range of additional features, including calcinosis, lipodystrophy, treatment-resistant rash and internal organ involvement. Patients can have heterogeneous responses to treatment, which also poses a challenge for clinical management. At present there are few reliable predictive biomarkers for response to treatment.

Historically, disease courses in JDM have been described as monocyclic, polycyclic or chronic typically based on clinical assessment within the first 2 years after diagnosis [2–6]. Longer-term cross-sectional follow-up studies have indicated that some patients have ongoing disease activity and damage [7–14], underscoring the need to collect and understand data on long-term outcomes in JDM beyond the first 2 years. Longitudinal analyses enable insight into outcomes that change over time, and can be used to characterize a disease course in a data-driven manner. An example is a recent study that applied longitudinal methods to understand disease evolution using data from 95 patients at The Hospital for Sick Children, Toronto, Canada [15].

Growth mixture models (GMMs) are a longitudinal method for identifying latent or ‘hidden’ subgroups of patients with similar mean trajectories of disease activity over time when the number of groups and group membership are unknown [16]. In contrast to other methods for latent class trajectory analysis, GMMs allow individuals within each class to have differences in their trajectories. Although these alternative approaches are easier to implement, GMMs have a more substantive interpretation, as shown by the Canadian study results, which identified three different GMM ‘typical’ trajectories in total DAS, with baseline as DAS their predictor [15].

The aims of this study were to use GMM analysis to identify classes of patients with JDM with different trajectories of disease activity and to identify predictors of class membership at baseline, using data from a large, multicentre cohort study.

## Methods

### Patients and clinical data

All patients were recruited to the UK-wide JDM Cohort and Biomarker Study (JDCBS) [17], with ethical approval (UK Northern and Yorkshire Medical Research and Ethics Committee MREC/1/3/22). All patients provided written informed consent and age-appropriate assent. Patients are recruited at 16 centres in the UK (Supplementary Table S1, available at *Rheumatology* online), with de-identified patient data held within the JDCBS database. Centres are anonymized in this analysis to protect patient confidentiality. The JDCBS holds focus groups with young people with JDM to involve patients in study design and disseminates research at JDM regular family days.

For all patients (*n* = 519 at time of study), demographic data and clinical features at first-recorded visit were collected, including diagnosis, abnormal respiration, arthritis, cutaneous ulceration, lipodystrophy and calcinosis. Abnormal respiration was defined as ‘shortness of breath’, ‘accessory muscle use’, ‘tachypnea’, ‘requires oxygen’ or ‘ventilated’ [18]. All other clinical features were defined by the treating clinician at the first-recorded visit. For patients in whom autoantibodies were measured (*n* = 379), these data were generally generated using immunoprecipitation and ELISA as previously described [19–21], and by line blot for a limited number of cases. Median duration of follow-up was 5.1 (2.6–8.6) years. Clinic visits were recorded approximately twice per year (Supplementary Table S1, available at *Rheumatology* online).

At all recorded visits (*n* = 5306), several measures were collected including the physician’s global assessment (PGA) of global disease activity on a visual analogue scale and the cutaneous components of the modified DAS for assessing skin disease (comprising Gottron’s papules, heliotrope rash, vasculitis and erythema) [22]. The PGA in our analysis is a more subjective score that reflects cutaneous, musculoskeletal and other manifestations of JDM that influence the physician’s overall assessment of global disease activity, and may in some cases be influenced by features such as calcinosis that can be attributed to ongoing activity by some physicians, but disease damage by others. Higher levels of disease activity are indicated by higher scores in the PGA (range 0–10) and modified DAS (range 0–5) and lower scores of the Childhood Myositis Assessment Scale (CMAS) (range 0–52). For the modified DAS, when any of Gottron’s papules, heliotrope rash, vasculitis or erythema were missing they were treated as absent to avoid the need to impute this variable, similar to a previous analysis [18], although we recognize this approach may underestimate the true modified DAS. Since 11 patients never had a PGA recorded, the total number of patients when PGA was modelled as an outcome was 508.

Data are described using median and first and third quartiles for numeric variables (unless specified otherwise), and counts and percentages for categorical variables.

### Data exploration and identification of missing data patterns

The PGA and modified DAS longitudinal trajectories were described using spaghetti and lasagne plots. Follow-up ranged from 0.2 to 23.4 years, with very few observations being available after 10 years. For this reason follow-up was truncated at 10 years since diagnosis, in order to avoid including sparse observations that may be highly influential. This led to the inclusion of complete follow-up for 83.6% of patients (*n* = 4837 recorded visits) and a similar median length of follow-up of 4.9 (2.1–8.1) years in the truncated dataset.

Since for 13% of the available visits, PGA values were missing, patterns in missing data were explored. Data visualization did not indicate there were any patterns in the frequency of missing PGA over time or a relationship with duration of follow-up. Formal analyses of the pattern of missingness consisted of modelling the probability of missing a PGA observation during follow-up, accounting for clustering (as the same patient may have missing values at different visits) using generalized estimating equations (implemented with an exchangeable correlation matrix and robust standard errors). Potential predictors of missingness were: sex, age at diagnosis, time since diagnosis, baseline PGA and Centre, coded Centre A [*n* = 241 (46.4%)] *vs* the rest [*n* = 278 (53.6%), coded as ‘Other’]. Baseline PGA and Centre were found to be significantly associated with missingness. A random audit of all clinic forms received in a single month suggested clinical experience may influence whether PGA was missing; however, since allocation of patients to consultants or registrars is random, it was thought not to introduce bias.

### GMM analysis

GMMs are models for repeated observations of an outcome that allow individual trajectories to cluster within classes. These classes are unobserved (latent) but are inferred from the data. GMMs can be fitted to continuous outcomes like the PGA and modified DAS, assuming Gaussian or non-Gaussian distributions, and to categorical variables using ordered logistic regression models. We fitted GMMs to either the PGA or modified DAS as outcomes with time since diagnosis (years) having both fixed and random effects, while also allowing for class-specific effects of time (Supplementary Methods, available at *Rheumatology* online). Since exploratory analysis identified Centre as a predictor of missing PGA over time, a binary variable for Centre (Centre A or Other; Supplementary Table S3, available at *Rheumatology* online) was included in the GMM analysis of PGA in order to deal with the missing data patterns under the missing at random assumption. Although baseline PGA was also a predictor of missing PGA over time, it did not need to be included as it was already part of the outcome model, and therefore its incompleteness was dealt with by the maximum likelihood estimation, again under the missing at random assumption. Single class models were fitted initially to define the optimal transformations of outcome variables and best function of time, with models selected according to the lowest Bayesian information criterion value (Supplementary Table S4, available at *Rheumatology* online). A cubic term for time and square-root transformations of PGA and modified DAS gave the lowest Bayesian information criterion values.

Models with two to six classes were subsequently fitted. The optimal number of classes was determined by selecting the model with the lowest Bayesian information criterion and highest entropy (Supplementary Table S4, available at *Rheumatology* online), as recommended in [23]. Entropy is an indication of how well individuals have been allocated to each class on a scale of 0–1, with 1 meaning perfect classification, and is calculated using the mean posterior probability for each class. Posterior classification is also reported for each class for each GMM with two to six classes (Supplementary Table S4, available at *Rheumatology* online). The mean of posterior probabilities for each class is reported for each GMM with two to six classes (Supplementary Table S5, available at *Rheumatology* online).

### Sensitivity analyses

Sensitivity analyses were subsequently performed to identify whether there were differences in the results for males or females and to investigate whether results were influenced by late-entry individuals or by both late-entry individuals and individuals contributing fewer visits or by non-incident individuals (Supplementary Table S6, available at *Rheumatology* online). Two definitions of non-incident individuals were used: individuals recruited to the JDCBS later than a month since diagnosis (*n* = 184 excluded; the remaining cohort becoming the ‘inception cohort’), and individuals recruited to the JDCBS at least 5 years after diagnosis (*n* = 29). Individuals contributing few visits were defined as those contributing fewer than three visits (*n* = 79). These thresholds were selected according to the distributions of time from diagnosis to recruitment (years) and the numbers of visits.

### Lasso regression to predict class membership

Baseline predictors of membership of the classes predicted by GMM analysis were identified using Lasso regression, a regression method that involves automatic selection of predictors and regularization of estimated coefficients for improved prediction accuracy [24]. Lasso regression was performed using all clinical features recorded at the first visit as predictors: diagnosis, PGA, CMAS, modified DAS, age at diagnosis, time from diagnosis to first visit, and the presence of any of arthritis, abnormal respiration, calcinosis, lipodystrophy and ulceration. Myositis-specific autoantibodies (MSAs) were not included as a predictor due to missing data. Since 0–34% of data were missing at first-recorded visit for some clinical features, multiple imputation was first performed using baseline clinical features and demographic features to generate 10 imputed datasets.

For Lasso regression, the following steps were performed separately for each of these 10 imputed datasets (Supplementary Methods, available at *Rheumatology* online). In an initial step, 10-fold cross-validation was run to select the optimal value for the tuning parameter lambda, a parameter that contributes to the shrinkage penalty applied to the regression coefficients. Within each cross-validation, the area under the curve was used to select the value of lambda that minimized the cross-validation error. These lambda values were then used to fit the regression models and estimate the coefficients for the predictors retained across each imputed dataset.

To account for uncertainty in class assignment during the GMM stage of the analysis, observations were weighted by the probability of class membership for each individual patient, as predicted by the GMM. Where significant predictors were identified by the Lasso regression, the model was re-run with interaction terms between those predictors. Finally, the estimates for predictors with non-zero coefficients were pooled from each imputed dataset. Standard errors are not reported as these are unreliable for penalized regression [25]. Lasso regression analysis was repeated using deviance and misclassification error as the loss functions to select the value of lambda that minimized cross-validation error. The same predictors were identified using these loss functions, and similar estimates of effect size were obtained.

### Software

All analyses were performed using R version 3.4.4 [26]. GMM analyses were performed using the lcmm() function in the ‘lcmm’ package [27]. Lasso regression analyses were performed using the ‘glmnet’ package [28]. Code for GMM and Lasso regression analyses are given in Supplementary Methods, available at *Rheumatology* online. Multiple imputation was performed using the ‘mice’ package [29]. Generalized estimating equations were fitted using the geeglm() function in the ‘geepack’ package [30]. Lasagne plots were generated using the ‘longCatEDA’ package [31].

## Results

### Demographic, clinical and serological features of whole JDCBS cohort

For the whole JDCBS cohort (*n* = 519), most patients were female, white and had a diagnosis of definite JDM (Table 1). Almost half of the cohort were recruited at Centre A, with remaining patients recruited at 15 other centres (Supplementary Table S2, available at *Rheumatology* online). Median age at diagnosis was 7.7 years and median first-recorded PGA and modified DAS were 3.0 and 3, respectively. The most prevalent MSAs were anti-TIF1γ (16.9%) and anti-NXP2 (15.6%).

**Table 1:** Demographic, clinical and serological features of the UK Juvenile Dermatomyositis Cohort and Biomarker Study Cohort (*n* = 519)

Feature	
Contributing centre^a^, n (%)	
Centre A	241 (46.4)
Centre B	43 (8.3)
Centre C	42 (8.1)
Centre D	37 (7.1)
Centre E	29 (5.6)
Centre F	28 (5.4)
Centre G	27 (5.2)
Centre H	26 (5.0)
Centre I	12 (2.3)
Centre J	9 (1.7)
Centre K	8 (1.5)
Centre L	6 (1.2)
Centre M	5 (1.0)
Centre N	4 (0.8)
Centre O	1 (0.2)
Centre P	1 (0.2)
Sex, *n* (%)	
Female	364 (70.1)
Male	155 (29.9)
Ethnicity, *n* (%)	
White	402 (77.4)
Black	47 (9.1)
South Asian	33 (6.4)
Other	37 (7.1)
Diagnosis, *n* (%)	
Definite JDM	383 (73.8)
Probable JDM	46 (8.9)
Definite juvenile polymyositis	8 (1.5)
Probable polymyositis	2 (0.4)
JDM overlap with scleroderma	28 (5.4)
JDM overlap with systemic lupus erythematosus	5 (1.0)
JDM overlap with chronic polyarthritis	6 (1.2)
JDM overlap with mixed connective tissue disease	9 (1.7)
Polymyositis overlap with scleroderma	3 (0.6)
Polymyositis overlap with systemic lupus erythematosus	1 (0.2)
Mixed connective tissue disease	13 (2.5)
Focal myositis	6 (1.2)
Other idiopathic inflammatory myopathy	9 (1.7)
Age at diagnosis, 1st and 3rd quartile, years	7.7 (4.8–11.0)
Age at onset, median (IQR), years	6.9 (4.1–10.1)
Time since diagnosis, median (IQR), years	0.2 (0.1–1.1)
Baseline PGA^b^, median (IQR)	3.0 (1.0–5.9)
Baseline CMAS^c^, median (IQR)	42 (25.5–50)
Baseline modified DAS, median (IQR)	3 (1–4)
Baseline clinical feature^d^, *n* (%)	
Arthritis	114 (24.4)
Abnormal respiration	45 (9.2)
Calcinosis	40 (11.7)
Lipodystrophy	30 (6.3)
Ulceration	59 (12.5)
Autoantibody^e^, *n* (%)	
No-detectable autoantibody	87 (23.0)
Unknown bands	68 (17.9)
Anti-TIF1γ	64 (16.9)
Anti-NXP2	59 (15.6)
Anti-MDA5	23 (6.1)
Anti-PmScl	22 (5.8)
Anti-Mi2	14 (3.7)
Anti-U1RNP	12 (3.2)
Anti-SRP	9 (2.4)
Anti-HMGCR	4 (1.1)
Anti-SAE	3 (0.8)
Anti-Jo1	3 (0.8)
Anti-Ro	2 (0.5)
Anti-PL-12 and Anti-Ro52	2 (0.5)
Anti-Ku	2 (0.5)
Anti-U3RNP	1 (0.3)
Anti-U1RNP and Anti-TIF1γ	1 (0.3)
Anti-Topo	1 (0.3)
Anti-PL-7	1 (0.3)
Anti-Mi2 and Anti-NXP2	1 (0.3)

a Contributing centres have been allocated codes to protect patient confidentiality.

b Complete data on PGA at baseline available for 87.2% of cases.

c Complete data on CMAS at baseline available for 76.6% of cases.

d Complete data available as follows: arthritis (90%), abnormal respiration (94.2%), calcinosis (66.1%), lipodystrophy (91.3%), ulceration (91.1%).

e Of the total *n* = 379 on whom autoantibody data were available, *n* = 364 (96%) were analysed by immunoprecipitation and confirmed by ELISA and *n* = 15 (4%) were analysed by lineblot. CMAS: Childhood Myositis Assessment Scale; IQR: interquartile range; PGA: physician’s global assessment.

**Fig. 1:**
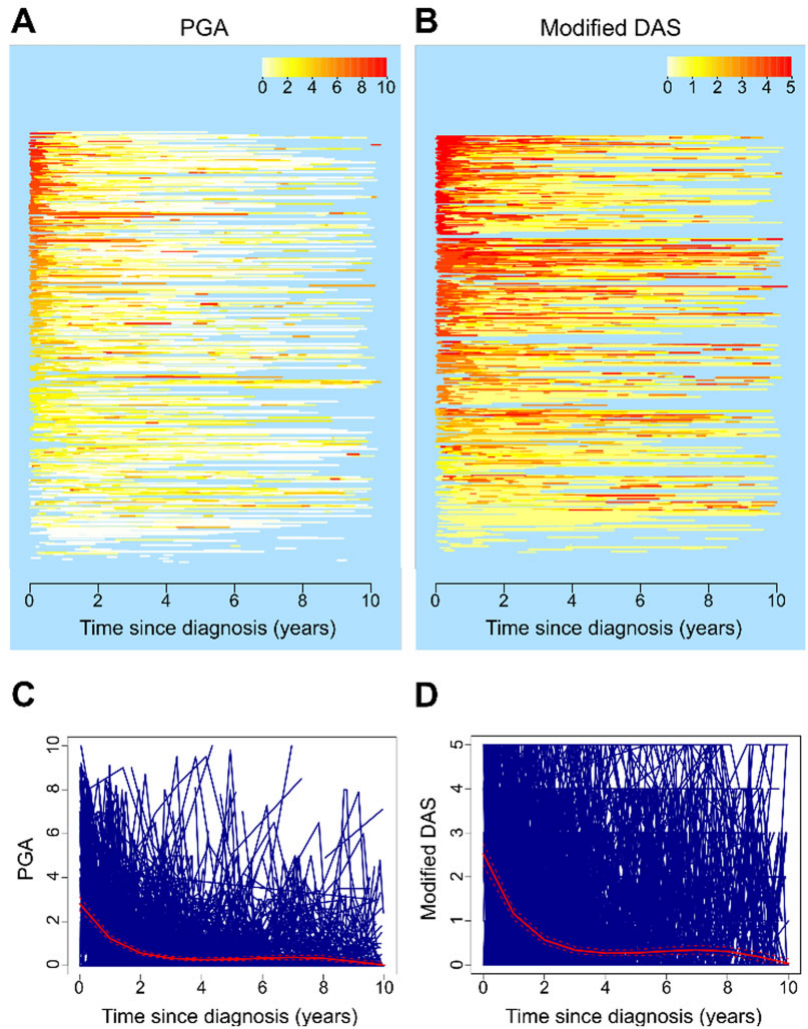
Longitudinal trajectories of JDM patients up to 10 years post-diagnosis (**A**, **B**) Lasagne plots for PGA (**A**) and modified DAS (**B**). Each horizontal line represents an individual patient, with red colours representing high disease activity. (**C**, **D**) Spaghetti plots for PGA (**C**) and modified DAS (**D**). Each blue line represents the disease trajectory of an individual patient and the red line represents the median trajectory. PGA: physician’s global assessment.

Median (range) follow-up was 5.1 (0.2–23.4) years before follow-up time was truncated at 10 years, and 4.9 (2.6–8.1) years after truncation. Median time from diagnosis to first-recorded visit was 0.2 (0.1–1.1) years. Visual representation of patients’ longitudinal trajectories for up to the first 10 years post-diagnosis for both PGA and modified DAS for skin disease indicated that disease activity on average reduced to lower levels over time, although patterns were heterogeneous between patients (Fig. 1).

### Two-class GMM for PGA

**Fig. 2:**
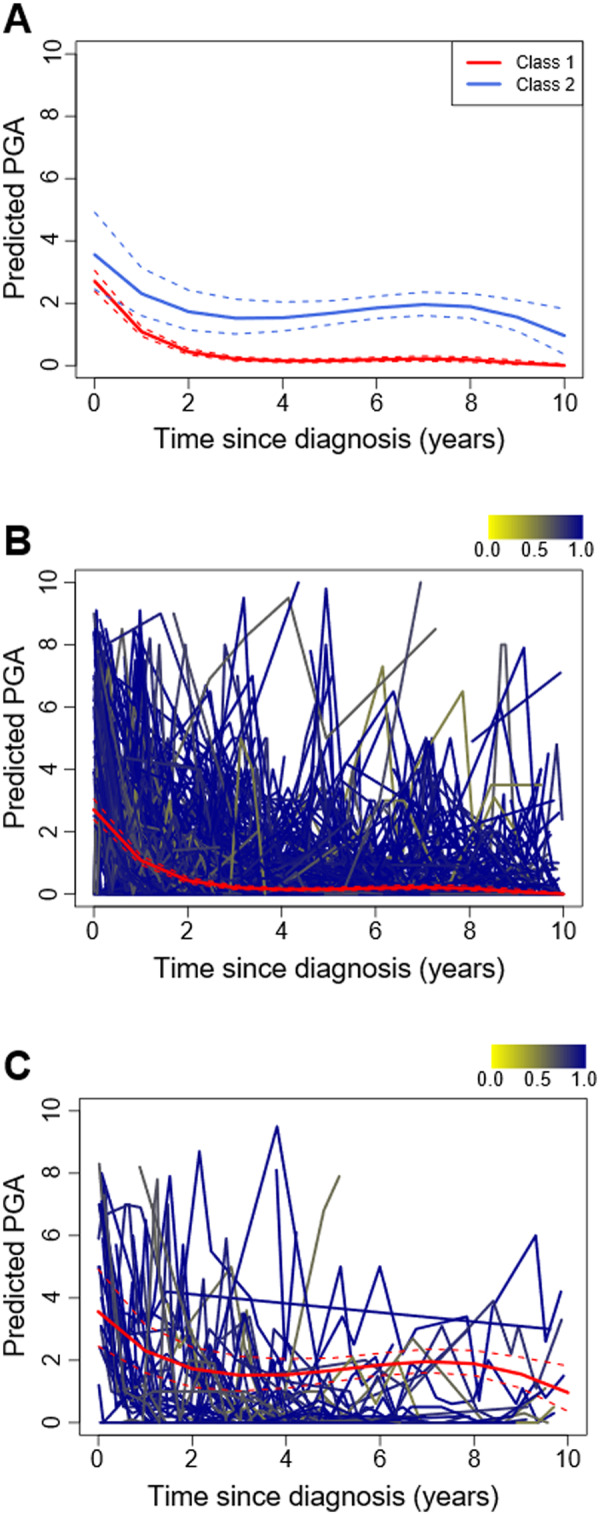
Two-class growth mixture model for global disease activity (**A**) Predicted median PGA (solid line) and 95% CI (dashed lines) for both classes. (**B**, **C**) Individual trajectories classified into class 1 (**B**) or class 2 (**C**), overlaid with the predicted median PGA (red solid line) and 95% CI (red dashed lines). Individual trajectories are coloured according to the posterior probability of class membership. PGA: physician’s global assessment.

To separate this heterogeneity into groups with more homogeneous patterns in disease trajectory, GMM analysis was applied. In the model fitted for PGA (*n* = 508), the two-class solution was preferred (Fig. 2A, Supplementary Fig. S1A and B, available at *Rheumatology* online). Most patients were predicted to belong in class 1 (*n* = 450, 89%). This group had slightly lower levels of global disease activity at diagnosis, which steadily decreased over time (Fig. 2A and B). By contrast, a small proportion of patients were predicted to belong in class 2 (*n* = 58, 11%), with their trajectories starting with slightly higher PGA levels and displaying more fluctuations over time (Fig. 2B and C).

Similar proportions of males and females and the major ethnic groups were represented in the two classes (Table 2). Higher proportions of patients in class 2 were diagnosed at recruitment with JDM overlap with scleroderma or other idiopathic inflammatory myopathy. Ages at diagnosis and symptom onset were similar across classes. Although baseline PGA was higher in patients in class 2, baseline modified DAS scores were similar. Although in general MSA proportions were similar across classes, a higher proportion of patients in class 2 had anti-NXP2 autoantibodies and a higher proportion of patients in class 1 had no-detectable autoantibody.

**Table 2:** Characteristics of patients assigned to the classes identified by GMMs for global and skin disease activities

	GMM for global disease activity		GMM for skin disease activity		
	(*n* = 508)^a^		(*n* = 519)^b^		
	Class 1	Class 2	Class 1	Class 2	Class 3
	(*n* = 450, 89%)	(*n* = 58, 11%)	(*n* = 81, 16%)	(*n* = 63, 12%)	(*n* = 375, 72%)
Sex, *n* (%)					
Female	315 (70.0)	40 (69.0)	50 (67.6)	47 (82.4)	232 (68.0)
Male	135 (30.0)	18 (31.1)	24 (32.4)	10 (17.5)	109 (32.0)
Ethnicity, *n* (%)					
White	349 (77.6)	43 (74.1)	53 (71.6)	51 (89.5)	265 (77.7)
Black	38 (8.4)	8 (13.8)	7 (9.5)	2 (3.5)	32 (9.4)
South Asian	28 (6.2)	5 (8.6)	9 (12.2)	3 (5.3)	19 (5.6)
Other	35 (7.8)	2 (3.4)	5 (6.8)	1 (1.8)	25 (7.3)
Diagnosis, *n* (%)					
Definite JDM	337 (74.9)	38 (65.5)	57 (77.0)	44 (77.2)	253 (74.2)
Probable JDM	41 (9.1)	4 (6.9)	4 (5.4)	4 (7.0)	33 (9.7)
Definite or probable polymyositis	9 (2.0)	1 (1.7)	0 (0)	0 (0)	5 (1.5)
JDM overlap with scleroderma	20 (4.4)	7 (12.1)	2 (2.7)	5 (8.8)	15 (4.4)
JDM overlap with mixed connective tissue disease	8 (1.8)	1 (1.7)	2 (2.7)	0 (0)	7 (2.1)
JDM overlap with chronic polyarthritis	6 (1.3)	0 (0)	2 (2.7)	1 (1.8)	3 (0.9)
JDM overlap with systemic lupus erythematosus	5 (1.1)	0 (0)	0 (0)	0 (0)	4 (1.2)
Other idiopathic inflammatory myopathy	24 (5.3)	7 (12.1)	7 (9.5)	3 (5.3)	21 (6.2)
Age at diagnosis, median (IQR), years	7.7 (4.7–11.0)	8.2 (5.1–10.8)	8.9 (5.5–10.5)	8.1 (3.9–11.7)	7.5 (4.8–11.2)
Age at onset, median (IQR), years	7.1 (4.2–10.1)	6.0 (3.7–9.5)	7.8 (4.5–10.1)	6.9 (3.1–9.9)	6.8 (4.2–10.3)
Time since diagnosis, median (IQR), years	0.2 (0.1–1.0)	1.1 (0.2–3.8)	0.2 (0.1–1.4)	0.6 (0.1–1.8)	0.2 (0.1–1.1)
Baseline PGA, median (IQR)	2.7 (1.0–5.4)	4.2 (2.8–6.7)	3.1 (2.0–6.7)	2.3 (1.0–4.4)	2.9 (1.0–5.9)
Baseline CMAS, median (IQR)	42 (26.5–51)	40 (19–47)	41 (26–51)	45 (39–52)	42 (24.3–50)
Baseline modified DAS, median (IQR)	3 (1–5)	3 (0.3–4)	4 (2–5)	3 (2–4)	3 (1–4)
Major myositis-specific autoantibody groups^c^, *n* (%)					
No-detectable autoantibody	82 (24.8)	5 (10.4)	11 (25.0)	10 (33.3)	63 (38.4)
Anti-TIF1γ	55 (16.6)	9 (18.8)	17 (38.6)	6 (20.0)	39 (23.8)
Anti-NXP2	49 (14.8)	10 (20.8)	9 (20.4)	8 (26.7)	39 (23.8)
Anti-MDA5	22 (6.6)	1 (2.1)	5 (11.4)	4 (13.3)	14 (8.5)
Anti-Mi2	13 (3.9)	1 (2.1)	2 (4.5)	2 (6.7)	9 (5.5)
Cross-tabulation with GMM for global disease activity, *n* (%)					
Class 1	—	—	61 (83.6)	46 (80.7)	300 (89.6)
Class 2	—	—	12 (16.4)	11 (19.3)	35 (10.4))
Cross-tabulation with GMM for skin disease activity, *n* (%)					
Class 1	61 (15.0)	12 (20.7)	—	—	—
Class 2	46 (11.3)	11 (19.0)	—	—	—
Class 3	300 (73.7)	35 (60.3)	—	—	—

Characteristics are calculated using the most likely class for each patient, as predicted by the relevant GMM.

a *n* = 508 individuals with PGA recorded at ≥1 visit.

b *n* = 519 individuals with no missing data for modified DAS.

c Percentages represent the proportion of patients with each of the listed autoantibody within each class for the relevant GMM on whom autoantibody data were available (autoantibody data available for *n* = 379 patients). GMM: growth mixture model; IQR: interquartile range.

### Three-class GMM for skin modified DAS

**Fig. 3:**
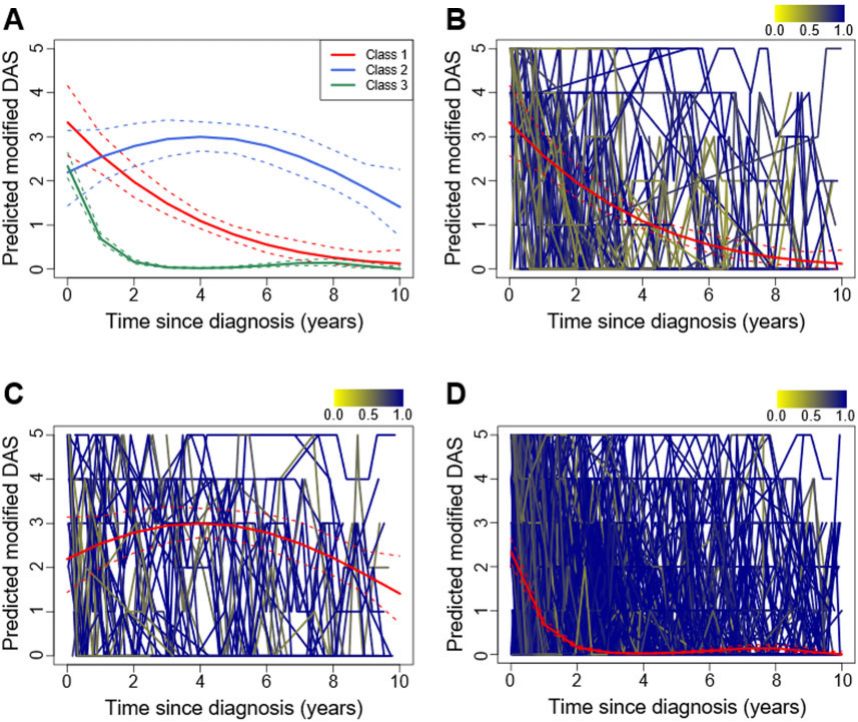
Three-class growth mixture model for skin disease activity (**A**) Predicted median modified DAS (solid line) and 95% CI (dashed lines) for the three classes. (**B**–**D**) Individual trajectories classified into class 1 (**B**), class 2 (**C**) or class 3 (**D**), overlaid with the predicted median modified DAS (red solid line) and 95% CI (red dashed lines). Individual trajectories are coloured according to the posterior probability of class membership.

A GMM was also fitted for skin modified DAS (*n* = 519), with a three-class solution being preferred (Fig. 3A, Supplementary Fig. S1C and D, available at *Rheumatology* online). Patients who were predicted as belonging to class 1 (*n* = 81 16%) had high levels of skin disease activity at diagnosis, which on average reduced slowly over time (Fig. 3B). Patients in class 2 (*n* = 63, 12%), on the other hand, also had high levels of skin disease activity at diagnosis, which tended to remain high over time (Fig. 3C). Most patients were predicted to belong in class 3 (*n* = 375, 72%), with lower levels of skin disease activity at diagnosis that reduced more quickly over time (Fig. 3D).

Class 2 had slightly higher proportions of patients who were female, white and who were diagnosed at recruitment with JDM overlap with scleroderma (Table 2). Ages at diagnosis and symptom onset, and baseline scores for PGA, CMAS and modified DAS were similar across all three classes. Similar to the GMM fitted for PGA, proportions of MSA were similar across classes, but class 1 had a higher proportion of patients with anti-TIF1γ autoantibodies.

Cross-tabulation of the two-class GMM for global disease activity with the three-class GMM for skin disease activity indicated a higher proportion (19%) of patients in class 2 of the GMM fitted to PGA were in class 2 of the GMM fitted to modified DAS than the in the other classes (respectively 16% and 10%, bottom of Table 2). Thus the closer association between these two classes indicates a joint ongoing disease activity for both PGA and modified DAS.

### Sensitivity analyses

Sensitivity analyses for the results obtained on both outcomes indicated that coefficients, the overall shape of predicted trajectories and class proportions did not change substantially in models fitted with females only, males only, restriction to the inception cohort and exclusion of late-entry individuals, and exclusion of both late-entry individuals and individuals contributing few visits (Supplementary Table S5, available at *Rheumatology* online).

### Predictors of PGA classes

**Fig. 4:**
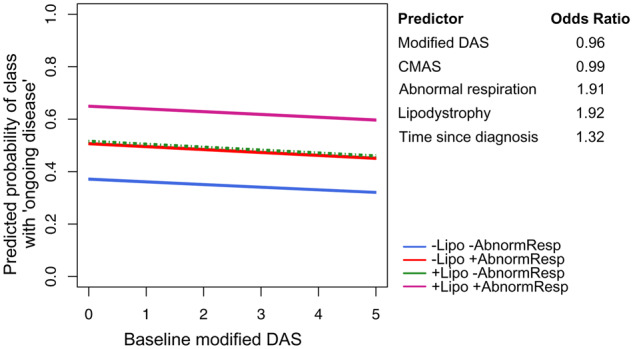
Predicted probabilities and ORs of belonging to global disease activity class 2 (‘ongoing disease activity’) The figure shows the predicted probabilities for each combination of modified DAS, lipodystrophy and abnormal respiration. Median baseline values for CMAS and time since diagnosis were used to calculate predicted probabilities. The table on the right reports the estimated ORs in the final model. Standard errors are not reported as these are unreliable for penalized regression [25]. The predicted probabilities for lipodystrophy only obscure those for abnormal respiration only, due to similar estimated ORs for these two predictors. CMAS: Childhood Myositis Assessment Scale; OR: odds ratio.

Predictive analyses of class membership were performed using the classification obtained from the GMM for PGA, as a measure of disease activity that captures multiple features of this multi-system disease. Indeed, the GMM for global disease activity had a higher entropy than the GMM for skin disease activity, and hence better classification of patients. Baseline predictors of membership in class 2 of the GMM for PGA were identified using Lasso regression, a method that involves automatic selection of predictors and regularization of coefficients, as described above. Baseline modified DAS, CMAS, abnormal respiration, lipodystrophy and time from diagnosis to first-recorded visit were identified as significant predictors of class 2 membership, with mutually adjusted estimated odds ratios of 0.96, 0.99, 1.91, 1.92 and 1.32, respectively. The modification of the association with abnormal respiration due to lipodystrophy corresponding to these interactions is visualized in Fig. 4.

None of these baseline variables were retained by the Lasso when this analysis was performed to identify possible predictors of class membership for the GMM for modified DAS.

## Discussion

This study represents the largest GMM analysis in JDM (*n* = 519) with this length of follow-up, and the first of a nationwide multicentre study. Our model of global disease activity identified two different trajectories of disease activity over time. While most patients (89%) had lower levels of disease activity that improved over time, a subset of patients (11%) had higher levels of baseline disease activity and tended to have more ongoing severe disease. Baseline variables that predicted whether patients belonged to this latter class identified by GMM were abnormal respiration, lipodystrophy and time since diagnosis. As a secondary analysis, trajectories of skin disease activity were modelled using GMM. While most patients (72%) had lower levels of skin disease that improved over time, smaller groups had high levels of skin disease that improved over time (16%), and high levels of skin disease that remained high over time (12%). A higher proportion of patients in class 2 of the GMM fitted to PGA, which had more ongoing severe disease, were in class 2 of the GMM fitted to modified DAS, which was similarly characterized by ongoing skin disease activity. Sensitivity analyses performed on subsets of the cohort participants did not indicate any systematic bias induced by the inclusions of possibly heterogeneous individuals. Separate analyses by sex also did not indicate any strong dissimilarities in the trajectories or class probabilities.

GMM analysis may represent a useful approach for defining sub-phenotypes of JDM, to complement current approaches for defining sub-phenotypes by MSAs, by focusing on disease outcomes. Interestingly, the major MSAs were distributed similarly across the GMM-defined classes, which could suggest the typologies identified by these models are different from the clinical features that characterized MSA-defined sub-phenotypes. Sub-phenotypes defined by GMM are driven by data on outcomes. Furthermore, the potential to weight individuals by the probability of class membership in subsequent analyses allows some accommodation of uncertainty in class allocation. A strength of GMM and other latent class methods, in contrast to alternative clustering approaches, is that they are model-based, with objective criteria for selecting models.

Our findings complement the work reported by Lim *et al.* [15] that identified three classes based on modelling the total DAS across over 40 months. In that analysis (*n* = 95) there were two larger classes, one with lower levels of disease activity at baseline that reduced slightly (42%), one with higher levels of disease activity that reduced to the lowest levels of disease activity (55%), and a much smaller third class with the highest levels of baseline disease activity that remain high over time (3%). Interestingly, our work on an independent cohort is consistent with this previous study, with the larger class resulting from the GMM analysis of PGA (class 1) paralleling the two larger classes in the Canadian analysis combined, as the classes that show improvement over time. It may be that this difference in number of classes between the studies reflects greater numbers of patients included, a longer duration of follow-up and use of a different (but related) outcome measure. In their analysis, the total DAS is a standardized score based on three specific cutaneous and three specific musculoskeletal findings [32], whereas the PGA in our analysis is a more subjective score that reflects cutaneous, musculoskeletal and other manifestations of JDM that influence the physician’s overall assessment of global disease activity, and may in some cases be influenced by features such as calcinosis that can be attributed to ongoing activity by some physicians, but disease damage by others.

This study has limitations. While *n* = 519 is a large sample size for a rare disease, our findings need to be validated independently before they can be generalized to the general population of patients with JDM. Nonetheless, in this regard the similarities with the independent Canadian study are encouraging. A further limitation arises from missing data. Although we sought to account for missing data patterns, we may not have achieved this perfectly. While it would have been useful to identify groups with similar muscle disease activities over time, there were too many missing CMAS values to do so. Although 96% of our autoantibody data were generated using the same method, inconsistent testing methods are a challenge for combining data [33, 34]. Future work could investigate how medication exposure both influences and is influenced by disease trajectories. While underlying differences in patient-mix were adjusted for in our GMM for PGA by including an indicator for Centre A (which contributed to 46% of patients), future work might investigate whether any of these differences might affect disease trajectories.

In summary, we have shown that GMM analysis can identify sub-phenotypes of JDM based on longitudinal disease outcomes, including global disease activity. Future work may identify biological differences in these classes, which could form the basis for a stratified approach to treatment in JDM.

*Funding*: This research was supported by the NIHR Biomedical Research Centre at Great Ormond Street Hospital for Children NHS Foundation Trust (GOSH).

*Disclosure statement*: The authors have declared no conflicts of interest.

## Supplementary data

Supplementary data are available at *Rheumatology* online.

## Supplementary Material

keaa497_Supplementary_Data
