# Supplementary material for: Identification and prediction of novel classes of long-term disease trajectories for patients with juvenile dermatomyositis using growth mixture models
Source: Rheumatology (Oxford). 2020 Nov 4;60(4):1891–901. doi: 10.1093/rheumatology/keaa497 (PMC8023987; doi:10.1093/rheumatology/keaa497)
Supplement: keaa497_Supplementary_Data [file keaa497_supplementary_data.zip › keaa497-suppl_data/rhe-20-0326-File006.docx]

**Supplementary Methods:**

**R code used to fit GMM using the “lcmm” package**

Single class model where “sqrtPGA” is the square root-transformed outcome for physician’s global disease activity and a cubic term for time is used, which is also fitted as a random effect. “Centre” represents a binary variable for Centre A or Other. “Patient.Code” represents the unique patient identification code. “dat” represents the dataframe containing the longitudinal dataset.

m1n <- lcmm(sqrtPGA ~ poly(TimeSinceDiagnosis, degree = 3, raw = TRUE) + Centre, subject = "Patient.Code",

random = ~ poly(TimeSinceDiagnosis, degree = 3, raw = TRUE),

data = dat,

ng = 1)

For the 2-class model, the cubic term for time was modelled as a fixed effect, a random effect and a mixture (i.e. class-specific fixed effects). The gridsearch option allows 100 sets of initial values generated randomly, using as initial values the estimates from the single-class model. A maximum of 20 iterations for each of these values was used.

m2n <- gridsearch(

lcmm(sqrtPGA ~ **poly(TimeSinceDiagnosis, degree = 3, raw = TRUE)** + Centre,

**mixture = ~ poly(TimeSinceDiagnosis, degree = 3, raw = TRUE)**,

subject = "Patient.Code",

**random = ~ poly(TimeSinceDiagnosis, degree = 3, raw = TRUE)**,

data = dat,

ng = 2),

rep = 100,

maxiter = 20,

minit = m1n)

This fits a GMM specified as follows:

*Z_ij|c_ =* $\beta_{0i}^{c}$ + $\beta_{1i}^{c}$*t_j_* + $\beta_{2}^{c}$*Centre +* $\varepsilon_{ij}^{c}$, for *c* = 1, 2

where

- *Z_ij|c_* represents the *j*th observation of PGA at time-point *t_j_* for individual *i*, given the membership of individual *i* in class *c* (where c=1,2)
- $\beta_{0i}^{c}$ = $\beta_{0}^{c}$ + $u_{0i}^{c}$ i.e. the individual-specific intercept for class *c* in the reference centre A, comprising a fixed component ($\beta_{0}^{c}$) and a random component ($u_{0i}^{c}$)
- $\beta_{1i}^{c}$ = $\beta_{1}^{c}$ + $u_{1i}^{c}$ i.e. the individual-specific coefficient for time for class *c*, comprising a fixed component ($\beta_{1}^{c}$) and a random component ($u_{1i}^{c}$)
- $\beta_{2}^{c}$ represents the coefficient for Centres other than A and class *c*, comprising a fixed component ($\beta_{1}^{c}$) only (i.e. no individual-specific effects)
- $u_{i}^{c}$= ($u_{0i}^{c}, u_{1i}^{c}$ ) is assumed to follow a joint Normal distribution with covariance matrix with non-zero off-diagonal elements reflecting departures for individual *i* from the population average trajectory for class *c* (where that trajectory for class *c* is defined by $\beta_{0}^{c}+\beta_{1}^{c}tj$)
- $\varepsilon_{ij}^{c}$ represents the residual error i.e. the distance between the observed and the true PGA for class *c* at time-point *t_j_*

**R code used to fit Lasso regression using the “glmnet” package**

Define the model matrix and outcome:

modmatrix <- model.matrix(pga.class ~ . -1, data=dat)

outcome <- (dat$pga.class)

Cross-validation to define the optimal value of lambda:

cv.lasso <- cv.glmnet(x=modmatrix, y=outcome, nfolds=10, family="binomial", alpha=1, type.measure="auc")

Fit the model:

fit <- glmnet(x=modmatrix, y=outcome, family="binomial", alpha=1, lambda=cv.lasso$lambda.min)

**Supplementary Table S1: Frequency of data-points by contributing centre**

| **Contributing Centre** | **Frequency of Visits Per Patient** | **Median Interval Between Visits Per Patient (years)** |
| --- | --- | --- |
| Centre A | 12 [6-17] | 0.34 [0.29-0.48] |
| Centre B | 9 [3.5-10.5] | 0.37 [0.25-0.50] |
| Centre C | 10 [6-17] | 0.34 [0.27-0.47] |
| Centre D | 7 [4-10] | 0.34 [0.27-0.45] |
| Centre E | 5 [4-9] | 0.63 [0.46-0.93] |
| Centre F | 8 [6-10.25] | 0.52 [0.37-0.81] |
| Centre G | 7 [2.5-10] | 0.50 [0.38-0.68] |
| Centre H | 3.5 [2-5] | 0.46 [0.29-0.64] |
| Centre I | 3.5 [2-7.25] | 0.49 [0.35-0.84] |
| Centre J | 6 [1-10] | 0.39 [0.31-0.56] |
| Centre K | 5.5 [4.75-9] | 0.33 [0.29-0.50] |
| Centre L | 5.5 [3.25-7.75] | 0.15 [0.11-0.50] |
| Centre M | 4 [4-6] | 0.48 [0.34-0.67] |
| Centre N | 4 [3.25-4] | 0.5 [0.49-0.53] |
| Centre O | 5 | 0.34 |
| Centre P | 7 | 0.25 |

**Supplementary Table S2: Comparison of demographic and baseline clinical features between patients treated at Centre A versus other centres**

| **Feature** | **Centre A (n=241)** | **Other centres combined (n=278)** |
| --- | --- | --- |
| **Sex**  Female  Male | 167 (69.3%)  74 (30.7%) | 197 (70.9%)  81 (29.1%) |
| **Ethnicity**  White  Black  South Asian  Other | 169 (70.1%)  39 (16.2%)  15 (6.2%)  18 (7.5%) | 233 (83.8%)  8 (2.9%)  18 (6.5%)  19 (6.8%) |
| **Diagnosis**  Definite JDM  Probable JDM  Definite juvenile polymyositis  Probable polymyositis  JDM overlap with scleroderma  JDM overlap with systemic lupus erythematosus  JDM overlap with chronic polyarthritis  JDM overlap with mixed connective tissue disease  Polymyositis overlap with scleroderma  Polymyositis overlap with systemic lupus erythematosus  Mixed connective tissue disease  Focal myositis  Other idiopathic inflammatory myopathy | 165 (68.5%)  31 (12.9%)  2 (0.8%)  0 (0.0%)  18 (7.5%)  0 (0.0%)  5 (2.1%)  3 (1.2%)  1 (0.4%)  0 (0.0%)  5 (2.1%)  4 (1.7%)  7 (2.9%) | 218 (78.4%)  15 (5.4%)  6 (2.2%)  2 (0.7%)  10 (3.6%)  5 (1.8%)  1 (0.4%)  6 (2.2%)  2 (0.7%)  1 (0.4%)  8 (2.9%)  2 (0.7%)  2 (0.7%) |
| **Age at diagnosis** (years) | 7.4 [4.4-10.3] | 8.0 [5.2-11.4] |
| **Age at onset** (years) | 6.4 [3.8-9.5] | 7.3 [4.6-11.0] |
| **Baseline PGA^a^** | 3.5 [1.7-6.3] | 2.4 [0.5-5.0] |
| **Baseline CMAS^b^** | 41 [19-50] | 43 [33-51] |
| **Baseline modified DAS^c^** | 4 [2-5] | 3 [0-4] |

^a^Baseline PGA values missing for 10.4% of values for Centre A and 18.3% for other centres combined

^b^Baseline CMAS values missing for 19.5% of values for Centre A and 27.7% for other centres combined

**Supplementary Table S3: Univariate GEE models to predict patterns of missingness of PGA over time**

| **Predictor in univariate model** | **Estimate** | **95% Confidence Interval** | **P-value** |
| --- | --- | --- | --- |
| **Centre^a^**  Centre I  Centre C  Centre D  Centre H  Centre E  Centre F  Centre B  Centre G | 1.39  0.41  1.15  0.93  0.54  1.60  0.81  2.07 | 0.59-3.30  0.30-0.71  0.76-1.76  0.48-1.80  0.29-1.01  0.99-2.58  0.55-1.18  1.38-3.10 | 0.46  0.00043  0.51  0.83  0.054  0.056  0.28  0.00042 |
| **Male sex^b^** | 0.85 | 0.68-1.06 | 0.16 |
| **Age at diagnosis** | 1.02 | 0.99-10.5 | 0.12 |
| **First-recorded PGA^c^** | 1.00 | 0.97-1.04 | 0.84 |
| **Missing first-recorded PGA** | 3.48 | 2.80-4.32 | <2e-16 |
| **Clinical experience^d^**  Registrar  Research nurse | 33.0  2.0 | 2.19-1406.0  0.26-41.6 | 0.025  0.56 |

^a^Centre A as reference category. Centres contributing fewer patients were excluded from this analysis

^b^Female sex as reference category

^c^Note there are 78/533 patients with missing first-recorded PGA. These patients were excluded in this analysis and a separate analysis investigated whether missing first-recorded PGA predicts having a missing PGA over time

^d^Consultant as reference category (of person who submitted the clinic visit form)

**Supplementary Table S4: Metrics for GMM models fit for global disease and skin disease activities**

| **Outcome** | **Transformations** | **Number of groups** | **BIC** | **Entropy^1^** | **Class 1%** | **Class 2%** | **Class 3%** | **Class 4%** | **Class 5%** | **Class 6%** |
| --- | --- | --- | --- | --- | --- | --- | --- | --- | --- | --- |
| **PGA** | Linear outcome | 1 | 16289.43 |  | 100.0 |  |  |  |  |  |
|  | Log outcome | 1 | 18335.87 |  | 100.0 |  |  |  |  |  |
|  | Sqrt outcome | 1 | 8789.17 |  | 100.0 |  |  |  |  |  |
|  | Quadratic term for time, sqrt outcome | 1 | 8424.82 |  | 100.0 |  |  |  |  |  |
|  | Cubic term for time, sqrt outcome | 1 | 8265.38 |  | 100.0 |  |  |  |  |  |
|  | Cubic term for time, sqrt outcome | 2 | 8245.27 | 0.73 | 88.6 | 11.4 |  |  |  |  |
|  | Cubic term for time, sqrt outcome | 3 | 8259.39 | 0.55 | 8.3 | 88.8 | 3.0 |  |  |  |
|  | Cubic term for time, sqrt outcome | 4 | 8268.16 | 0.41 | 7.5 | 5.1 | 64.4 | 23.0 |  |  |
|  | Cubic term for time, sqrt outcome | 5 | 8295.06 | 0.22 | 14.6 | 7.5 | 17.3 | 54.5 | 6.1 |  |
|  | Cubic term for time, sqrt outcome | 6 | 8337.44 | 0.15 | 12.0 | 0.8 | 10.8 | 71.1 | 5.1 | 0.2 |
| **Modified DAS** | Linear outcome | 1 | 17711.38 |  | 100.0 |  |  |  |  |  |
|  | Log outcome | 1 | 22362.26 |  | 100.0 |  |  |  |  |  |
|  | Sqrt outcome | 1 | 11039.59 |  | 100.0 |  |  |  |  |  |
|  | Quadratic term for time, sqrt outcome | 1 | 10765.88 |  | 100.0 |  |  |  |  |  |
|  | Cubic term for time, sqrt outcome | 1 | 10662.47 |  | 100.0 |  |  |  |  |  |
|  | Cubic term for time, sqrt outcome | 2 | 10602.91 | 0.76 | 85.0 | 15.0 |  |  |  |  |
|  | Cubic term for time, sqrt outcome | 3 | 10564.55 | 0.64 | 15.8 | 67.1 | 17.1 |  |  |  |
|  | Cubic term for time, sqrt outcome | 4 | 10570.26 | 0.48 | 17.5 | 15.4 | 11.8 | 55.3 |  |  |
|  | Cubic term for time, sqrt outcome | 5 | 10570.64 | 0.42 | 40.3 | 12.7 | 11.2 | 18.7 | 17.1 |  |
|  | Cubic term for time, sqrt outcome | 6 | 10573.51 | 0.30 | 9.8 | 28.9 | 13.9 | 4.6 | 14.1 | 28.7 |

^1^ Entropy is calculated by taking the sum over the K classes of the product (class probability*log(class probability)), then dividing by log(K), and then adding 1.

**Supplementary Table S5: Mean posterior probabilities for each class** **for GMM models fit for global disease and skin disease activities with at least 2 classes**

| **Outcome** | **Number of groups in model** | **Class** | **Probability 1** | **Probability 2** | **Probability 3** | **Probability 4** | **Probability 5** | **Probability 6** |
| --- | --- | --- | --- | --- | --- | --- | --- | --- |
| PGA | 2 | Class 1  Class 2 | 0.9258  0.1247 | 0.0742  0.8753 | - | - | - | - |
|  | 3 | Class 1  Class 2  Class 3 | 0.8116  0.0371  0.1700 | 0.1172  0.8892  0.0856 | 0.0712  0.0737  0.7444 | - | - | - |
|  | 4 | Class 1  Class 2  Class 3  Class 4 | 0.8503  0.0774  0.0112  0.0637 | 0.0470  0.6993  0.1149  0.0523 | 0.0046  0.1039  0.7609  0.1515 | 0.0982  0.1194  0.1130  0.7325 | - | - |
|  | 5 | Class 1  Class 2  Class 3  Class 4  Class 5 | 0.6639  0.0914  0.1441  0.0403  0.0694 | 0.0682  0.8492  0.0266  0.0109  0.0742 | 0.1909  0.0100  0.5733  0.1576  0.1141 | 0.0374  0.0018  0.1700  0.6998  0.0787 | 0.0396  0.0476  0.0861  0.0913  0.6636 | - |
|  | 6 | Class 1  Class 2  Class 3  Class 4  Class 5  Class 6 | 0.4715  0.0011  0.0133  0.1224  0.0718  0.4600 | 0.0175  0.8975  0.0098  0.0260  0.0003  0.0000 | 0.0034  0.0960  0.7739  0.0518  0.1091  0.0000 | 0.1852  0.0046  0.0913  0.7071  0.1444  0.0275 | 0.0607  0.0003  0.1080  0.0454  0.6558  0.0017 | 0.2616  0.0006  0.0037  0.0474  0.0186  0.5108s |
| Modified DAS | 2 | Class 1  Class 2 | 0.8903  0.0643 | 0.1097  0.9357 | - | - | - | - |
|  | 3 | Class 1  Class 2  Class 3 | 0.7909  0.1125  0.0704 | 0.0935  0.8678  0.0238 | 0.1156  0.0197  0.9058 | - | - | - |
|  | 4 | Class 1  Class 2  Class 3  Class 4 | 0.7691  0.0164  0.1223  0.1905 | 0.0238  0.8656  0.1002  0.0051 | 0.0783  0.1121  0.7597  0.0049 | 0.1288  0.0059  0.0178  0.7995 | - | - |
|  | 5 | Class 1  Class 2  Class 3  Class 4  Class 5 | 0.8168  0.0477  0.0710  0.0519  0.0137 | 0.0346  0.7238  0.0107  0.0961  0.1727 | 0.0725  0.0247  0.8489  0.0966  0.0026 | 0.0701  0.0643  0.0645  0.7348  0.0035 | 0.0060  0.1395  0.0049  0.0206  0.8074 | - |
|  | 6 | Class 1  Class 2  Class 3  Class 4  Class 5  Class 6 | 0.6685  0.1560  0.1884  0.0259  0.0063  0.0266 | 0.1399  0.6224  0.0114  0.0420  0.0046  0.0883 | 0.1469  0.0477  0.7926  0.0055  0.0023  0.0075 | 0.0270  0.0559  0.0053  0.8353  0.0865  0.0311 | 0.0044  0.0322  0.0011  0.0325  0.8446  0.0882 | 0.0133  0.0858  0.0011  0.0587  0.0557  0.7583 |

**Supplementary Table S6. Sensitivity analyses for GMMs fit for global disease and skin disease activities**

|  |  | **Class** | **N(%)** | **Fixed effect coefficient for intercept** | **Fixed effect coefficient for time (linear term)** | **Fixed effect coefficient for time (quadratic term)** | **Fixed effect coefficient for time (cubic term)** | **Coefficient for Centre “Other” (PGA outcome only)** |
| --- | --- | --- | --- | --- | --- | --- | --- | --- |
| PGA (2 classes, sqrt outcome, cubic time) | Selected model | 1 | 450 (88.6%) | 0 | -1.32931 | 0.24726 | -0.01445 | -0.09222 |
|  |  | 2 | 58 (11.4%) | 0.435 | -0.83718 | 0.1819 | -0.01146 |  |
|  | Males only | 1 | 132 (86.3%) | 0 | -1.5769 | 0.31961 | -0.01983 | -0.26014 |
|  |  | 2 | 21 (13.7%) | -0.10785 | -0.59538 | 0.13317 | -0.00827 |  |
|  | Females only | 1 | 39 (11.0%) | 0 | -0.95056 | 0.20769 | -0.01322 | -0.05793 |
|  |  | 2 | 316 (89.0%) | -0.58196 | -1.23494 | 0.21806 | -0.01229 |  |
|  | Exclusion of late-entry individuals (> 5 years) | 1 | 432 (89.8%) | 0 | -1.3274 | 0.24494 | -0.01434 | -0.10029 |
|  |  | 2 | 49 (10.2%) | 0.47229 | -0.95134 | 0.21667 | -0.01408 |  |
|  | Exclusion of late-entry individuals and individuals contributing few visits (>3 visits) | 1 | 380 (89.2%) | 0 | -1.33537 | 0.24653 | -0.01443 | -0.09852 |
|  |  | 2 | 46 (10.8%) | 0.50584 | -1.01888 | 0.23147 | -0.01496 |  |
|  | Exclusion of prevalent individuals (time from diagnosis to first recorded visit > 1 month) | 1 | 15 (8.4%) | 0 | -1.10463 | 0.22664 | -0.01422 | -0.02873 |
|  |  | 2 | 164 (91.6%) | -0.77712 | -1.50577 | 0.29605 | -0.01786 |  |
| Modified DAS (3 classes, sqrt outcome, cubic time) | Selected model | 1 | 81 (15.6%) | 0 | -0.3615 | 0.01147 | 0.00009 | - |
|  |  | 2 | 64 (12.1%) | -0.56057 | 0.20613 | -0.02619 | 0.00009 | - |
|  |  | 3 | 375 (72.3%) | -0.48199 | -1.37406 | 0.26655 | -0.01545 | - |
|  | With centred time variable | 1 | 348 (67.1%) | 0 | -0.2163 | 0.10077 | -0.01261 | - |
|  |  | 2 | 89 (17.2%) | -0.37222 | 0.01513 | 0.04743 | -0.00829 | - |
|  |  | 3 | 82 (15.8%) | 1.73754 | 0.02991 | 0.02022 | -0.00671 | - |
|  | Males only | 1 | 21 (13.6%) | 0 | 0.36895 | -0.06325 | 0.00262 | - |
|  |  | 2 | 110 (71.0%) | 0.16631 | -1.43564 | 0.27176 | -0.01486 | - |
|  |  | 3 | 24 (15.5%) | 0.27974 | -0.46371 | 0.07893 | -0.00613 | - |
|  | Females only | 1 | 93 (25.6%) | 0 | -0.00727 | -0.05278 | 0.00396 | - |
|  |  | 2 | 63 (17.3%) | -1.86289 | -0.6342 | 0.15234 | -0.01005 | - |
|  |  | 3 | 208 (57.1%) | 0.20072 | -1.61039 | 0.30871 | -0.01812 | - |
|  | Exclusion of late-entry individuals (> 5 years) | 1 | 49 (10.0%) | 0 | 0.25372 | -0.0408 | 0.00106 | - |
|  |  | 2 | 359 (73.3%) | 0.08602 | -1.37905 | 0.2687 | -0.01568 | - |
|  |  | 3 | 82 (16.7%) | 0.5617 | -0.35895 | 0.01114 | 0.00012 | - |
|  | Exclusion of late-entry individuals and individuals contributing few visits (>3 visits) | 1 | 79 (18.5%) | 0 | -0.37386 | 0.01495 | -0.00013 | - |
|  |  | 2 | 304 (71.2%) | -0.4493 | -1.39465 | 0.27055 | -0.01574 | - |
|  |  | 3 | 44 (10.3%) | -0.51735 | 0.22864 | -0.03749 | 0.00093 | - |
|  | Exclusion of prevalent individuals (time from diagnosis to first recorded visit > 1 month) | 1 | 123 (74.1%) | 0 | -1.44842 | 0.28277 | -0.01639 | - |
|  |  | 2 | 28 (16.9%) | 0.62233 | -0.18522 | -0.06432 | 0.00546 | - |
|  |  | 3 | 15 (9.0%) | -0.00127 | 0.31749 | -0.07235 | 0.00335 | - |

The Juvenile Dermatomyositis Research Group members were as follows: Dr Kate Armon, Mr Joe Ellis-Gage, Ms Holly Roper, Ms Vanja Briggs and Ms Joanna Watts (Norfolk and Norwich University Hospitals), Dr Liza McCann, Mr Ian Roberts, Dr Eileen Baildam, Ms Louise Hanna, Ms Olivia Lloyd, Susan Wadeson and Michelle Andrews (The Royal Liverpool Children’s Hospital, Alder Hey, Liverpool), Dr Phil Riley, Ms Ann McGovern, Verna Cuthbert (Royal Manchester Children’s Hospital, Manchester), Dr Clive Ryder, Mrs. Janis Scott, Mrs. Beverley Thomas, Professor Taunton Southwood, Dr Eslam Al-Abadi and Ruth Howman (Birmingham Children’s Hospital, Birmingham), Dr Sue Wyatt, Mrs Gillian Jackson, Dr Mark Wood, Dr Tania Amin, Dr Vanessa VanRooyen and Ms Deborah Burton, Louise Turner & Sarah Hanson (Leeds General Infirmary, Leeds), Dr Joyce Davidson, Dr Janet Gardner-Medwin, Dr Neil Martin, Ms Sue Ferguson, Ms Liz Waxman and Mr Michael Browne, Ms Roisin Boyle, Ms Emily Blyth (The Royal Hospital for Sick Children, Yorkhill, Glasgow), Dr Mark Friswell, Professor Helen Foster, Mrs Alison Swift, Dr Sharmila Jandial, Ms Vicky Stevenson, Ms Debbie Wade, Dr Ethan Sen, Dr Eve Smith, Ms Lisa Qiao, Mr Stuart Watson and Ms Claire Duong (Great North Children’s Hospital, Newcastle), Dr Helen Venning, Dr Rangaraj Satyapal, Mrs Elizabeth Stretton, Ms Mary Jordan, Dr Ellen Mosley, Ms Anna Frost, Ms Lindsay Crate, Dr Kishore Warrier, Stefanie Stafford, Kelly Sandhu & Tracey Dandy (Queens Medical Centre, Nottingham), Professor Lucy Wedderburn, Dr Clarissa Pilkington, Dr Nathan Hasson, Dr Muthana Al-Obadi, Dr Giulia Varnier, Dr Sandrine Lacassagne, Mrs Sue Maillard, Mrs Lauren Stone, Ms Elizabeth Halkon, Ms Virginia Brown, Ms Audrey Juggins, Dr Sally Smith, Mrs Sian Lunt, Ms Elli Enayat, Mrs Hemlata Varsani, Miss Laura Kassoumeri, Miss Laura Beard, Miss Katie Arnold, Mrs Yvonne Glackin, Ms Stephanie Simou, Dr Beverley Almeida, Dr Kiran Nistala, Dr Raquel Marques, Dr Claire Deakin, Dr Parichat Khaosut, Ms Stefanie Dowle, Dr Charalampia Papadopoulou, Dr Shireena Yasin, Dr Christina Boros, Dr Meredyth Wilkinson, Dr Chris Piper, Mrs Cerise Johnson-Moore, Ms Lucy Marshall, Ms Emily Robinson (Great Ormond Street Hospital, London), Dr Kevin Murray (Princess Margaret Hospital, Perth, Western Australia) Dr Coziana Ciurtin, Dr John Ioannou, Mrs Caitlin Clifford and Ms Linda Suffield (University College London Hospital, London) Ms Helen Lee, Ms Sam Leach, Ms Helen Smith, Dr Anne-Marie McMahon, Ms Heather Chisem, Jeanette Hall, Ruth Kingshott and Maxine Mutten (Sheffield’s Children’s Hospital, Sheffield); Dr Nick Wilkinson, Ms Emma Inness, Ms Eunice Kendall, Mr David Mayers, Ruth Etherton, Danielle Miller and Dr Kathryn Bailey (Oxford University Hospitals, Oxford); Dr Jacqui Clinch, Ms Natalie Fineman and Ms Helen Pluess-Hall and Suzanne Sketchley (Bristol Royal Hospital for Children, Bristol); Dr Joyce Davidson, Margaret Connon and Ms Lindsay Vallance (Royal Aberdeen Children’s Hospital); Dr Kirsty Haslam, Charlene Bass-Woodcock, Trudy Booth and Ms Louise Akeroyd (Bradford Teaching Hospitals); Dr Alice Leahy, Amy Collier, Rebecca Cutts, Emma Macleod, Dr Hans De Graaf, Dr Brian Davidson, Sarah Hartfree, Danny Pratt, Elizabeth Fofana and Lorena Caruana (University Hospital Southampton) and all the children, young people and their families who have contributed to this research.

**Supplementary figure legend**

**Supplementary Figure 1: Model metrics for GMMs fitted for global disease activity and skin disease activity.** (A) BIC and (B) entropy against number of classes for GMMs fitted for global disease activity. (C) BIC and (D) entropy against number of classes for GMMs fitted for skin disease activity. BIC: Bayesian information criterion; GMM: growth mixture model.
