# Supplementary figures and images for: Identification and prediction of novel classes of long-term disease trajectories for patients with juvenile dermatomyositis using growth mixture models
Source: Rheumatology (Oxford). 2020 Nov 4;60(4):1891–901. doi: 10.1093/rheumatology/keaa497 (PMC8023987; doi:10.1093/rheumatology/keaa497)

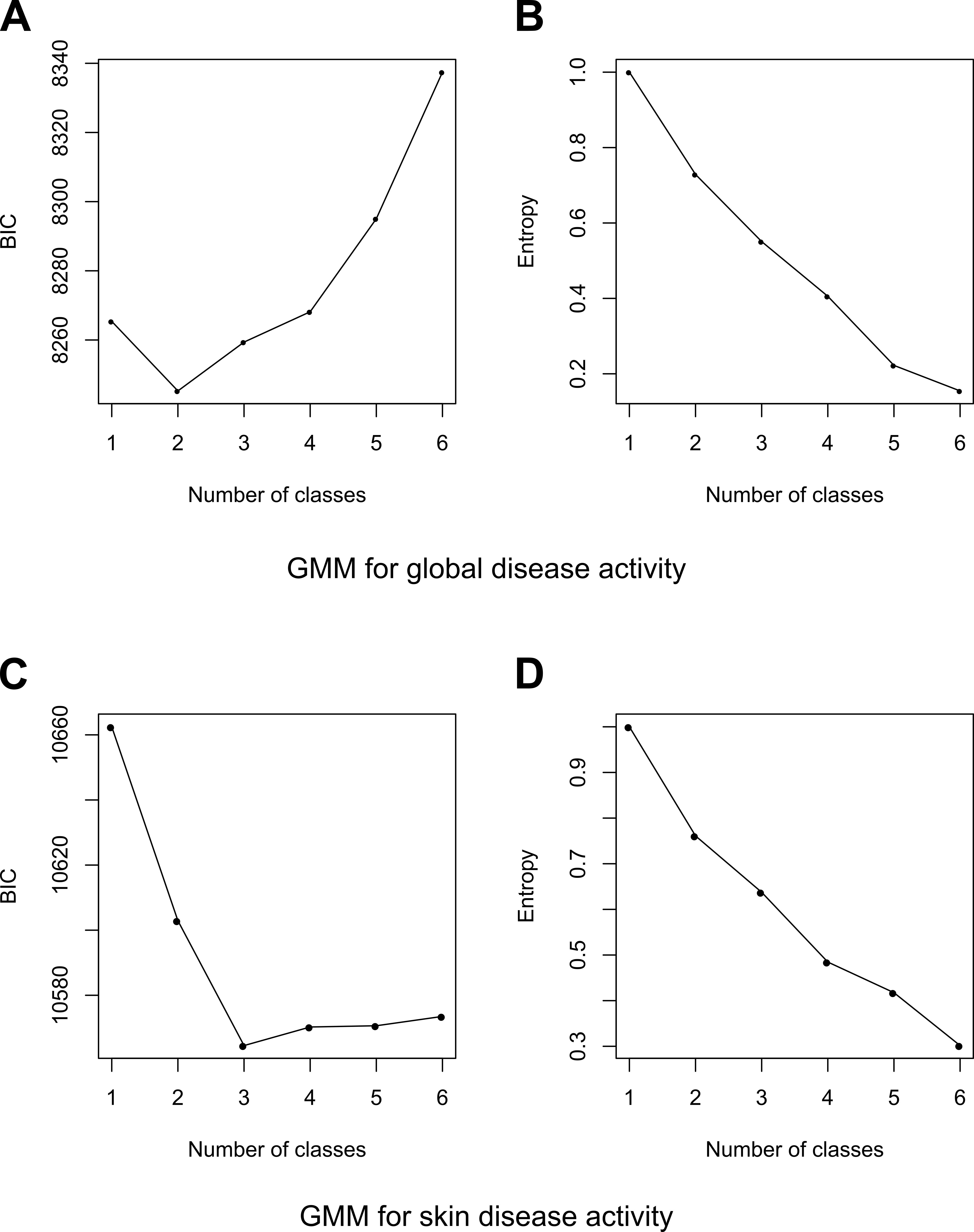

Supplement: keaa497_Supplementary_Data [file keaa497_supplementary_data.zip › keaa497-suppl_data/rhe-20-0326-File007.png]
